# Veterinary Journalism

**Published:** 1890-08

**Authors:** 


### EDITORIAL.

### VETERINARY JOURNALISM.

In ordinary journalism, from that of the daily newspapers to that of the various monthly periodicals treating of popular subjects or taking the place of novels and light literature, there are several factors which lead to success; the furnishing of material which will interest a large number of readers, cheapness of the publication, and advertisements are all important, but the latter by far the most so, and we find that the best papers are those whose advertising profits allow them to be liberal, even extravagant, in obtaining their reading matter.

A few journals of human medicine find financial success in large subscription lists, being able to supply the best products of medical writers to the very large number of educated physicians who are scattered all over the land, but the vast majority of medical journals find their support in the numerous advertisements of remedies of any and every nature, the most of which are used by an inferior class of practitioners much as the drugs of the old sea captains used to be, No. 1 for a cold, No. 2 for a colic, No. 3 for the fits, and if the action of the preparation was not understood, at least it was presumed that a poison had not been furnished.

The publications of branches of special science, for which there must necessarily be a limited number of readers, do not offer very great fields for any but *ad hoc* advertisers, and usually are supported by the societies of the science of which the subject matter treats, and are carried on as a luxurious necessity by those directly interested in the welfare of their calling. Veterinary journals are closely allied to the latter class. There are but a limited number of veterinarians who desire a journal of any kind, for we must eliminate at once from any consideration a large percentage of those found even on the registration lists in States, where such exist, as in Pennsylvania and New York, where large numbers are registered Sam his X mark Smith. Again, there are subscribers who write that the reading matter does not suit them; they "want prescriptions that will cure diseases," etc. All veterinary journalism in the United States has been, and is, a labor of love, and is carried on because those directly interested think it should be.

There are many, as the faculties of veterinary schools, and the members of active veterinary societies, who should be equally interested in aiding the editorial work, but who are not. The pages of The Journal of Comparative Medicine and Veterinary Archives are open to the profession in the United States and Canada, and offer it a medium for the communication of interesting and valuable veterinary literature. Communications sent should be in such shape as to be accurate and not require the imagination to fill out what the writer thought but did not state in his paper. The Journal reaches the alumni of every veterinary college in the country, and desires to publish all matter of general interest in regard to the various schools. It is the duty of the faculties of these schools to furnish such information, and, when we send for an account of the graduation of a class, not furnish us with a list of names with no addresses, and refer us to the daily papers of a city, several hundred miles away, for an account of the commencement exercises, not even sending us the papers; another college answers to questions, as to any changes or alterations which have taken place, by informing us that four assistants have been added to the teaching corps (no names), and gives us information in regard to building changes which were published in the Journal several months ago. The secretaries of veterinary societies frequently forget to say where or when the meeting which they report was held. We suggest the following outline as a guide for reporting society meetings:

Name of society, cycle of meeting, when and where held.

Character of attendance, names if desired.

Business matters which the society desires for record. Personalities are not desirable unless there is reason for them.

When officers are elected, their addresses.

Abstracts, showing the leading points of papers, with discussion, or the paper in full when the officers consider that it merits publication.

Individuals also have a duty to perform. No case of special interest should be allowed to pass unreported. If it cannot be used at once it will be properly filed to use when it will fit into the right place by itself, or with others of its kind.

